# Editorials

**Published:** 1897-04

**Authors:** 


### HOMŒOPATHIC PHYSICIAN,

A MONTHLY JOURNAL OF

HOMŒOPATHIC MATERIA MEDICA AND CLINICAL MEDICINE.

"If our school ever gives up the strict inductive method of Hahnemann, we are lost, and deserve only to be mentioned as a caricature in the history of medicine."—CONSTANTINE HERING.

Vol. XVII. APRIL, 1897. No. 4.

### EDITORIALS.

THE MEMBERS OF THE INTERNATIONAL HAHNEMANNIAN ASSOCIATION are again reminded that the Editor of this journal is Chairman of the Bureau of Materia Medica, and they are all urged to contribute something toward the improvement of our knowledge of drug action in time for the next meeting. Those who are *not* members are invited to contribute papers on pathogenesis. These papers should all be sent to the Editor at the office of this journal.

THE EDITORIALS ON MATERIA MEDICA.—The Editor regrets that the demands upon his time have again prevented his preparing any more notes from Dr. Lippe's lectures. He hopes, however, to be able to give a good selection from his accumulated memoranda in the number for May.

NOTICE.—The editor regrets that the excessive clerical work of the journal, together with the demands of his practice, has made a delay in the acknowledgment of many communications received. Correspondents are assured that answers to their letters will not be much longer delayed.

